# Evidence of Stem Cell Efficacy on Developmental and Functional Alterations in Craniofacial Diseases

**DOI:** 10.3390/jcm10020302

**Published:** 2021-01-15

**Authors:** Marco Tatullo, Gianrico Spagnuolo

**Affiliations:** 1 Department of Basic Medical Sciences, Neurosciences and Sense Organs—University of Bari “Aldo Moro”, 70124 Bari, Italy; marco.tatullo@uniba.it; 2 Department of Neurosciences, Reproductive and Odontostomatological Sciences, University of Naples Federico II, 80131 Naples, Italy

Stem cells have improved the treatment of several diseases. Their efficacy has been widely debated over the last few decades, but their contribution to medical research has constantly increased [1].

Recently, a pivotal study supported by the National Institute of Dental and Craniofacial Research (NIDCR), leaded by Prof. Yang Chai, investigated stem cell-based therapies for children with craniosynostosis. Authors demonstrated that Mesenchymal Stem Cells (MSCs) combined with hydrogel were able to regenerate functional cranial sutures previously undergone craniosynostosis, restoring not only skull anatomy but also neurocognitive functions in an animal model [2]. This study spreads light on the potential benefits deriving from stem cell therapy on both skull deformation and related brain dysfunction in a mouse model.

Several research groups have worked on the role of genetic and epigenetic factors on craniosynostosis development, confirming a pivotal role of TWIST-1 gene mutations in Saethre–Chotzen syndrome (SCS). The discovery of genetic and molecular pathways has increased attention on alternative treatments to surgery: the group of Prof. Stan Gronthos demonstrated that pharmacological targeting of Kdm6a/b genes activity may regulate aberrant osteogenesis by TWIST-1 mutant cranial suture mesenchymal progenitor cells via deregulation of epigenetic modifiers, thus preventing cranial prefusion and the related functional impairment [3]. On the other hand, the group headed by Prof. Rena D’Souza have highlighted the novel therapeutic applications of molecular, cellular and material-based strategies on human craniofacial diseases. Innovation in biomaterials science, the recent engineering-based therapeutic approaches and the bioactive scaffolds have been demonstrated to modulate molecular signaling networks relevant to modulate craniofacial development [4].

The use of stem cell therapy to reactivate functional sutures is a clinical breakthrough also in the light of the high incidence of resynostosis after complex cranial vault reconstruction. Nevertheless, an early combined surgical and regenerative approach to craniosynostosis, may reduce or definitely prevent several related dental and maxillofacial implications [5], in addition to avoiding the onset of cognitive disorders previously discussed.

The crosstalk among stem cell therapy and clinical outcomes has been investigated also in studies on muscular [6], neural [7], cardiac [8], dental [9,10] or other medical fields. Chai and colleagues [2] have applied stem cell therapy and tissue engineering to craniosynostosis, demonstrating on mice that regenerated sutures may repristinate skull physiology, reverting brain alterations related to cranial abnormalities; interestingly, regenerated sutures were found to create a kind of biological niche able to promote the homing of endogenous brain MSCs that migrated and sustained cranial homeostasis and repair.

Therefore, the results reached by Prof. Yang Chai and his group are a milestone that will promote novel less-invasive, stem cell-based therapeutic approaches that will benefit patients affected by craniofacial developmental disorder involving related brain and organs alterations. Overall, stem cell research has obtained an important achievement towards its future clinical application in minimally invasive therapeutic procedures.
